# Supplementary figures and images for: Eco-evo-devo implications and archaeobiological perspectives of trait covariance in fruits of wild and domesticated grapevines
Source: PLoS One. 2020 Nov 6;15(11):e0239863. doi: 10.1371/journal.pone.0239863 (PMC7647109; doi:10.1371/journal.pone.0239863)

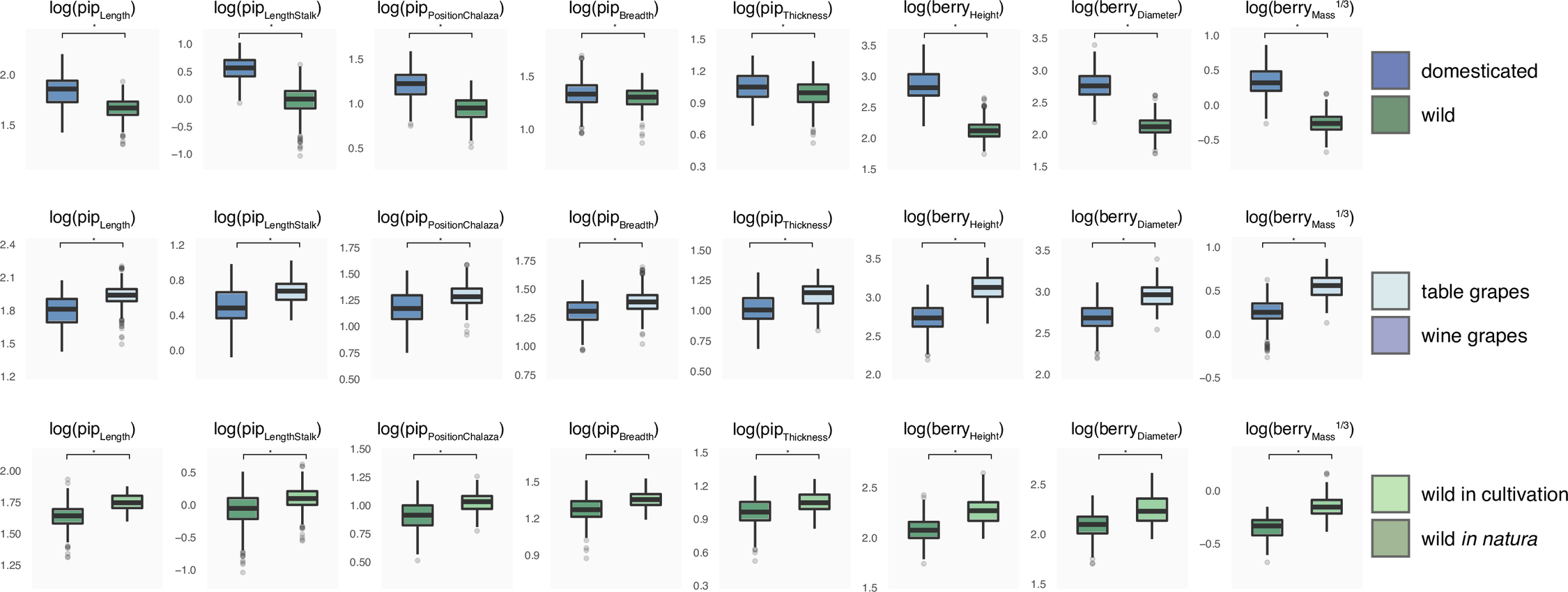

Supplement: S1 Fig — On rows are displayed different subsets: a) wild and domesticated grapevines, b) for domesticated accessions, table and wine varieties and, c) for wild accessions, those collected in natura and others cultivated as domesticated varieties. Different piposity levels are pooled (see Fig 3 for the detail). Differences are tested using Wilcoxon rank tests and all of them have a P<10−5. (TIF) [file pone.0239863.s001.tif]

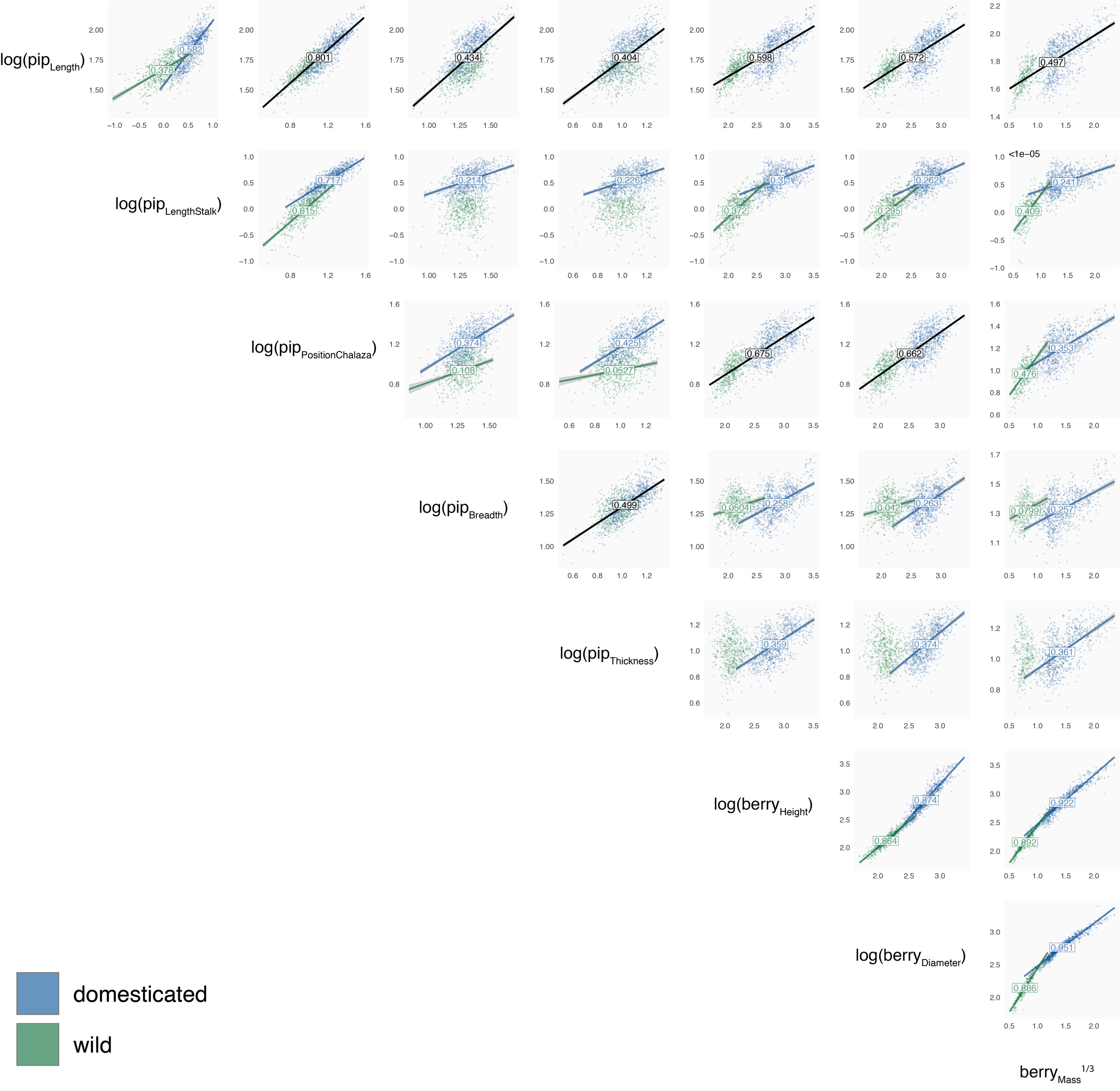

Supplement: S2 Fig — For the sake of readability, only the wild versus domesticated status are displayed using different colours (green for wild; blue for domesticated). If two regressions are justified, then they are shown using the corresponding colours; otherwise a single regression line is showed in black. Then, for each regression, the correlations are tested and, if significant, the adjusted R2 is displayed on the regression lines. (TIF) [file pone.0239863.s002.tif]

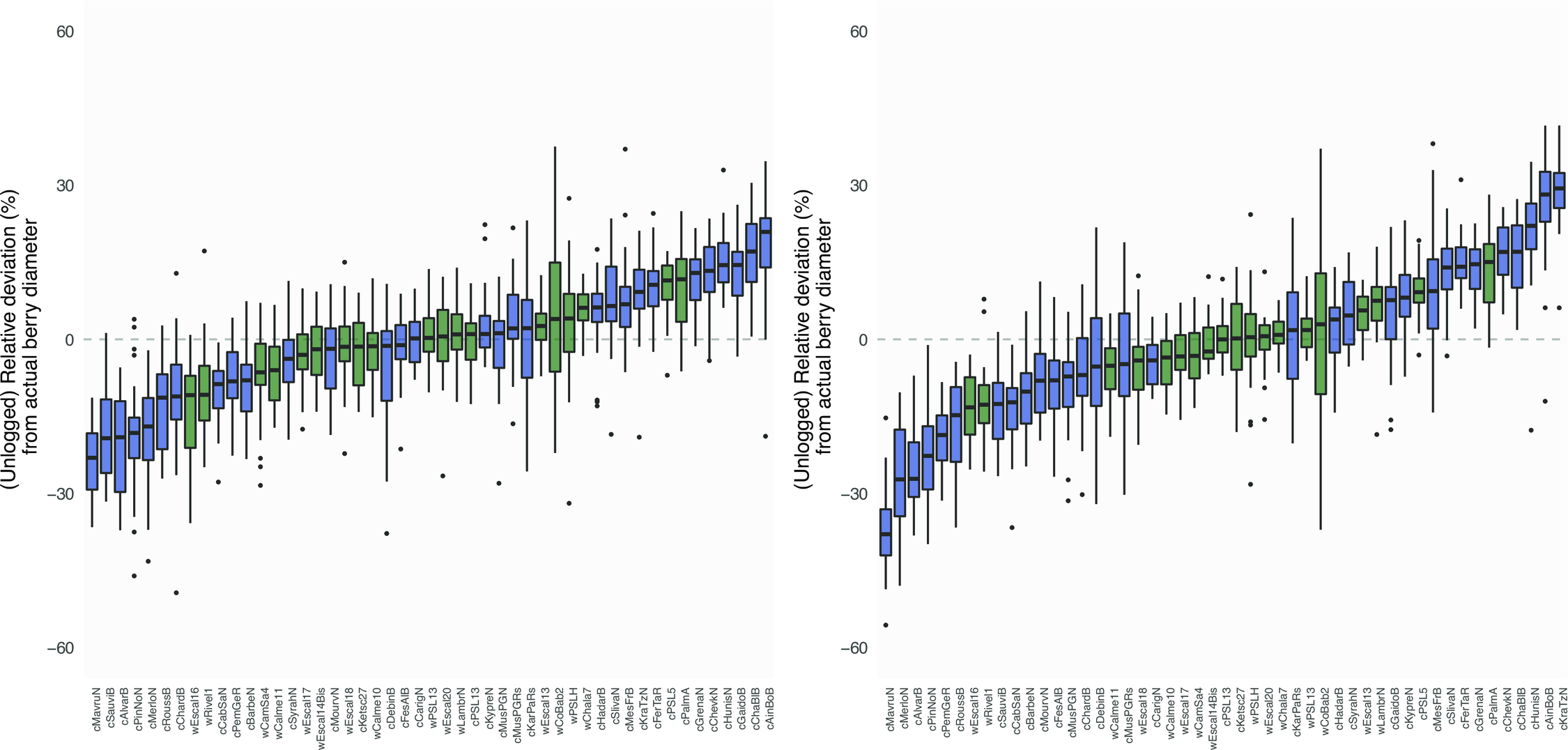

Supplement: S3 Fig — The relative deviation, at the accession level, and for unlogged measurements. Columns are for berry diameter and height, respectively. (TIF) [file pone.0239863.s003.tif]
