# Supplementary material for: Eco-evo-devo implications and archaeobiological perspectives of trait covariance in fruits of wild and domesticated grapevines
Source: PLoS One. 2020 Nov 6;15(11):e0239863. doi: 10.1371/journal.pone.0239863 (PMC7647109; doi:10.1371/journal.pone.0239863)
Supplement: Attachment — Submitted filename: Response to reviewers.docx [file pone.0239863.s004.docx]

Data availability:

>>> We made the datasets used in this study available in figshare. The reserved doi (doi:10.6084/m9.figshare.12696602) has been properly cited in the MS and will be active upon final acceptance. For now on, they can be accessed through this private link: https://figshare.com/s/959e803083001862ef3d

1. Is the manuscript technically sound, and do the data support the conclusions?

The manuscript must describe a technically sound piece of scientific research with data that supports the conclusions. Experiments must have been conducted rigorously, with appropriate controls, replication, and sample sizes. The conclusions must be drawn appropriately based on the data presented.

Reviewer #1: Yes

Reviewer #2: Partly

>>> We hope that the changes made now make the MS fully meet PLoS One standards.

4. Is the manuscript presented in an intelligible fashion and written in standard English?

Reviewer #1: Yes

Reviewer #2: No

>>> The MS has been written by a non-native (myself) but reviewed before the initial submission, and rereviewed now.

5. Review Comments to the Author

Please use the space provided to explain your answers to the questions above. You may also include additional comments for the author, including concerns about dual publication, research ethics, or publication ethics. (Please upload your review as an attachment if it exceeds 20,000 characters)

Reviewer #1: Although the authors have done good piece of research work providing the complete insight of changes occurred during the course of domestication of grapevine. But the length of manuscript need to shorten by removing of redundancy and should be presented in concise form.

Reviewer #2: The manuscript presents the effect of pip shape and size affecting size of grape berries. Though relevant but preliminary and also do not add much new to the existing knowledge. Experimentation lacks uniformity e.g., sampling from wild grown and cultural conditions thereby raises questions on varied effect of soil, climate, pruning intensity etc. Only a single year study

is insufficient to draw valid conclusions thus warrants comprehensive investigations. May be considered as a short communication than a full length paper. Language needs to be improved for more clarity and ambiguity be removed.

>>> The two reviewers agree about the length of the paper and asked to shorten it. We followed their advice and removed ~600 words (>12%) of the main MS body and did our best to keep it intelligible yet to make it more concise.

>>> The reviewers disagree about the novelty of the results brought here. The reviewer n¬∞2 stated that they "do not add much new to the existing knowledge".

We here disentangle many completely new aspects of the complex interplay between grapevine biology, development, and domestication using the pip and the berry forms as entry points. It is absolutely true that some long-known patterns (eg the shape difference between the wild and domesticated types) are not revealed but confirmed here, yet on a larger and more representative dataset. These totally new results presented here were presented as preliminary steps towards testing possible (and long lasted) biases in archaeobotanical inference such as the effect of the number of pips in a berry on their forms.

The reviewer n°2 also states that "sampling from wild grown and cultural conditions thereby raises questions on varied effect of soil, climate, pruning intensity etc.".

Differences indeed exist and that is exactly what we were looking for. For the analyzed traits, our results indicate that, half of what was supposed to be a "domestication gap" seems to be caused by cultivation.

We tempered a few statements but we have the feeling that the new questions brought by our findings are presented as promising perspectives, not definitive conclusions. It the Editor has the feeling our Discussion should be tempered once again, we will be happy to do it.

We hope that the changes made improve our manuscript and now fully satisfy both the reviewers and the editors.

Yours sincerely

Vincent Bonhomme
